# Effects of modifiable prehospital factors on survival after out-of-hospital cardiac arrest in rural versus urban areas

**DOI:** 10.1186/s13054-018-2017-x

**Published:** 2018-04-18

**Authors:** Wenche Torunn Mathiesen, Conrad Arnfinn Bjørshol, Jan Terje Kvaløy, Eldar Søreide

**Affiliations:** 1 Critical Care and Anesthesiology Research Group, Stavanger University Hospital, Stavanger, Norway; 2 Norwegian Air Ambulance Foundation, Department of Research and Development, Drøbak, Norway; 3 Department of Anesthesiology and Intensive Care, Stavanger University Hospital, Stavanger, Norway; 4 Department of Clinical Medicine, University of Bergen, Bergen, Norway; 5 Research Department, Stavanger University Hospital, Stavanger, Norway; 6 Department of Mathematics and Physics, University of Stavanger, Stavanger, Norway; 7 Stavanger University Hospital, Forskningens Hus, Armauer Hansensgate 2, P.O. box: 8100, 4068 Stavanger, Norway

**Keywords:** Out-of-hospital cardiac arrest, Rural, Urban, Survival, Cardiopulmonary resuscitation

## Abstract

**Background:**

The modifiable prehospital system factors, bystander cardiopulmonary resuscitation (CPR), emergency medical services (EMS), response time, and EMS physician attendance, may affect short- and long-term survival for both rural and urban out-of-hospital cardiac arrest (OHCA) patients. We studied how such factors influenced OHCA survival in a mixed urban/rural region with a high survival rate after OHCA.

**Methods:**

We analyzed the association between modifiable prehospital factors and survival to different stages of care in 1138 medical OHCA patients from an Utstein template-based cardiac arrest registry, using Kaplan-Meier type survival curves, univariable and multivariable logistic regression and mortality hazard plots.

**Results:**

We found a significantly higher probability for survival to hospital admission (OR: 1.84, 95% CI 1.43–2.36, *p* < 0.001), to hospital discharge (OR: 1.51, 95% CI 1.08–2.11, *p* = 0.017), and at 1 year (OR: 1.58, 95% CI 1.11–2.26, *p* = 0.012) in the urban group versus the rural group. In patients receiving bystander CPR before EMS arrival, the odds of survival to hospital discharge increased more than threefold (OR: 3.05, 95% CI 2.00–4.65, *p* < 0.001). However, bystander CPR was associated with increased patient survival to discharge only in urban areas (survival probability 0.26 with CPR vs. 0.08 without CPR, *p* < 0.001). EMS response time ≥ 10 min was associated with decreased survival (OR: 0.61, 95% CI 0.45–0.83, *p* = 0.002), however, only in urban areas (survival probability 0.15 ≥ 10 min vs. 0.25 < 10 min, *p* < 0.001). In patients with prehospital EMS physician attendance, no significant differences were found in survival to hospital discharge (OR: 1.37, 95% CI 0.87–2.16, *p* = 0.17). In rural areas, patients with EMS physician attendance had an overall better survival to hospital discharge (survival probability 0.17 with EMS physician vs. 0.05 without EMS physician, *p* = 0.019). Adjusted for modifiable factors, the survival differences remained.

**Conclusions:**

Overall, OHCA survival was higher in urban compared to rural areas, and the effect of bystander CPR, EMS response time and EMS physician attendance on survival differ between urban and rural areas. The effect of modifiable factors on survival was highest in the prehospital stage of care. In patients surviving to hospital admission, there was no significant difference in in-hospital mortality or in 1 year mortality between OHCA in rural versus urban areas.

## Background

Out-of-hospital cardiac arrest (OHCA) is a major cause of death in industrialized countries, and survival differs substantially, worldwide [1–3]. Survival is associated with certain OHCA patient factors, but these are generally non-modifiable [4]. However, some prehospital system factors in OHCA as bystander cardiopulmonary resuscitation (CPR), emergency medical services (EMS) response times, and the attendance of EMS physicians, are modifiable [4]. These factors represent opportunities for improvements in saving lives [4].

Bystander CPR is an essential intervention that increases the odds of survival of OHCA victims by two- to threefold [5]. As the effect of bystander CPR on survival declines rapidly over time, OHCA in remote areas with long EMS response times represent a particular challenge. Thus, a decrease in ambulance response times will lead to the annual saving of many additional lives [6]. In addition, some studies have shown that the attendance of an EMS physician is associated with improved survival [7, 8]. Thus, it is likely that bystander CPR, EMS response times, and the attendance of an EMS physician influence short- and long-term survival. Because some studies have shown that also population density is a predictor for survival, [9–11] the aim of this study is to examine how bystander CPR, EMS response time, and EMS physician attendance influence survival at different stages of care, and to what extent this differs between rural and urban areas.

## Methods

### Setting and design

The study region was that encompassed by the Stavanger Hospital Trust; it is situated on the southwest coast of Norway and constitutes 18 municipalities. The area covers 5700 km^2^ and spans both urban and rural municipalities. A minority of the population are inhabitants on islands that can only be reached by boat or helicopter. The cities of Stavanger and Sandnes and two other municipalities are the most densely populated areas, with over 200 inhabitants per km^2^ (218–1733 inhabitants per km^2^). Geographically, these four municipalities constitute the Stavanger peninsula that, for the purpose of this study, constitutes the urban area. The other 14 municipalities have populations of less than 200 inhabitants per km^2^ (1–148 inhabitants per km^2^). In the present study, these municipalities constitute the rural area.

The population of the study area increased from approximately 300,000 inhabitants to approximately 358,000 inhabitants during the study period, from 1 January 2006 to 31 December 2015 [12]. In order to categorize rural versus urban municipalities, we used the mean population density during the study period.

Stavanger University Hospital (SUH) is the only receiving hospital for OHCA patients in the study region. The Emergency Dispatch Centre (EDC) is responsible for coordinating 17 ambulance units allocated to eight ambulance stations, and one hospital-based, anesthesiologist-manned (EMS physician) rapid response unit that uses a helicopter for remote assignments, or a car for local assignments. The ambulance staff constitutes two crew members, of which at least one is an advanced life support-certified paramedic. In addition, the EDC dispatches general practitioners (GPs) on call in the local communities [13]. From October 2013, an EMS physician-manned fast response car was made available for a rescue helicopter team, but operates only in the closest proximity to the helicopter base. In addition, fire brigades equipped with automated external defibrillators are often dispatched by the EDC and operate as first responders.

The EDC has one nationwide alarm emergency telephone number (113). The dispatch is criteria-based (using the Norwegian index of emergency medical assistance). In cases of suspected cardiac arrest, the EDC instructs the caller to start CPR, including mouth-to-mouth resuscitation if the bystander is trained in CPR. In the case of untrained bystanders, the instruction is to carry out continuous chest compressions. The EDC also initiates a response by one or two ambulances, the EMS physician-manned rapid response unit and the local GP on call. The EDC aims to ensure at least one physician and two ambulance units at the scene. There were no systematic changes in the response pattern throughout the study period, except for the rapid response car of the rescue helicopter team. In cases where resuscitation did not lead to the return of spontaneous circulation (ROSC), the OHCA assignments were terminated at the scene or the patient was transported to hospital with ongoing resuscitation.

In Norway, training in basic CPR is provided through school systems, compulsory military service and voluntary organizations [14]. The oil industry employs a major work force in the Stavanger area, so CPR has been a part of health, environmental and safety training for many inhabitants in the study region, and there are also an increasing number of public access defibrillators (PADs). However, the EDC does not have alerting routines for PAD locations. As a part of post resuscitation intensive care at SUH, an established in-hospital treatment for all OHCA patients who have not regained consciousness after hospital admission is targeted temperature management and standardized post resuscitation care [15, 16]. Throughout the entire study period, emergency percutaneous coronary intervention was available to OHCA patients with ST-elevation myocardial infarction.

### Study population

**Fig. 1:**
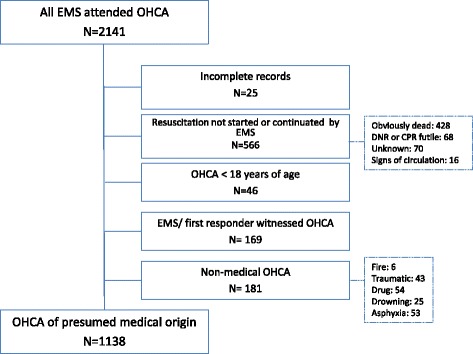
Inclusion of patients with medical cardiac arrest. *EMS* emergency medical services, *OHCA* out-of-hospital cardiac arrest

The local registry for all EMS-attended OHCA cases has been managed by SUH since 1996; Utstein template data are collected from the ambulance, the rapid response unit, and the EDC and cross checked [17]. The present study assessed all prospective datasets of individuals aged ≥18 years, collected between 1 January 2006 and 31 December 2014. OHCA missions for 2015 were collected from the Norwegian National Advisory Unit on Prehospital Emergency Medicine (NAKOS). We used the 2015 Utstein Resuscitation Registry Templates for Out-of-Hospital Cardiac Arrest to register the most likely primary cause of OHCA, which includes cardiac and other medical causes [18]. The following groups were excluded from further analysis: patients for whom no resuscitation attempts were made by the EMS, patients with EMS- or first responder-witnessed OHCA, patients with OHCA of non-medical origin, and cases with missing data. A total of 25 patients had achieved ROSC before EMS arrival, but with no registering of initial rhythm. Nine of these patients had been shocked prior to EMS arrival and were included. During the 10-year study period, 171 patients were admitted to hospital with ongoing resuscitation, of whom nine survived to discharge. Of 2141 EMS-attended OHCA incidents, a total of 1138 patients met the inclusion criteria for analysis (Fig. 1). Survival was measured at the following stages of care: hospital admission, ED discharge, 24 h after hospital admission, hospital discharge, and at 1 year. To further elucidate the relationship between prehospital modifiable factors and outcome, we also calculated the hazard of mortality between each stage.

### Study outcomes

The primary outcome was patient survival to hospital discharge. Secondary outcomes were survival to hospital admission, survival to emergency department (ED) discharge, survival to 24 h after hospital admission, and survival at 1 year after cardiac arrest.

### Study variables

The patient characteristic variables were age, gender, initial cardiac arrest rhythm (categorized as non-shockable [asystole, pulseless electrical activity] and shockable [ventricular fibrillation, pulseless ventricular tachycardia]), medical cause of cardiac arrest (cardiac and non-cardiac), ROSC (yes, no), location of cardiac arrest (at home, in public), municipality (urban, rural), whether the cardiac arrest was witnessed, not witnessed, CPR initiated (by bystanders [bystander defined as a person who is not responding as a part of an EMS]), first responders/EMS), EMS response time (the time interval in minutes from the call to the EDC until the first emergency vehicle arrived at the patient’s location) (< 10 min, ≥ 10 min), EMS physician attendance (yes, no), survival to hospital admission (yes, no), survival to ED discharge (yes, no), survival to 24 h after hospital admission (yes, no), survival to hospital discharge (yes, no), and survival at 1 year after cardiac arrest (yes, no).

### Statistical analysis

All data retrieved from the local hospital-managed OHCA registry were entered into a FilemakerPro6 database (Filemaker, Inc., Santa Clara, CA, USA) and via Microsoft Office®Excel 2010 (Microsoft Corporation, Redmond, WA, USA) to Statistical Package for the Social Sciences version 23 (IBM Corp, Armonk, NY,USA) and R version 3.3.2 [19] for statistical analysis and plotting. Continuous variables are reported as medians and interquartile ranges (IQR), categorical variables as numbers and percentages. The Mann-Whitney test was used to test for difference in the distribution of continuous variables between the urban and rural groups. Chi-square tests for independence (with Yates’ continuity correction) were used for assessing for differences in the distribution of categorical variables between groups. OHCA incidence rates were calculated, and age and gender adjusted according to the general Norwegian population, using data obtained from Statistics Norway [20]. Poisson regression analysis was used to test for differences in incidence rates between urban and rural areas. A Kaplan-Meier type plot was constructed to show estimated survival probabilities from after OHCA to five consecutive stages of care: Hospital admission, ED discharge, 24 h after admission, hospital discharge, and at 1 year. By focusing on these discrete stages of care rather than clock time we are able to do a detailed analysis of which factors are of importance for reaching these milestones. To further study the impact over time, hazard plots were also constructed. These plots show the estimated mortality probability between two consecutive stages of care, given survival to the start of the first stage. Chi-square tests were used to test for differences in mortality or hazards between groups for each stage of care. Logistic regression analysis was used to study the impact of urban and rural areas and other factors on survival to the different stages of care and at 1 year. In all tests, *p* values of < 0.05 were considered statistically significant.

## Results

**Fig. 2:**
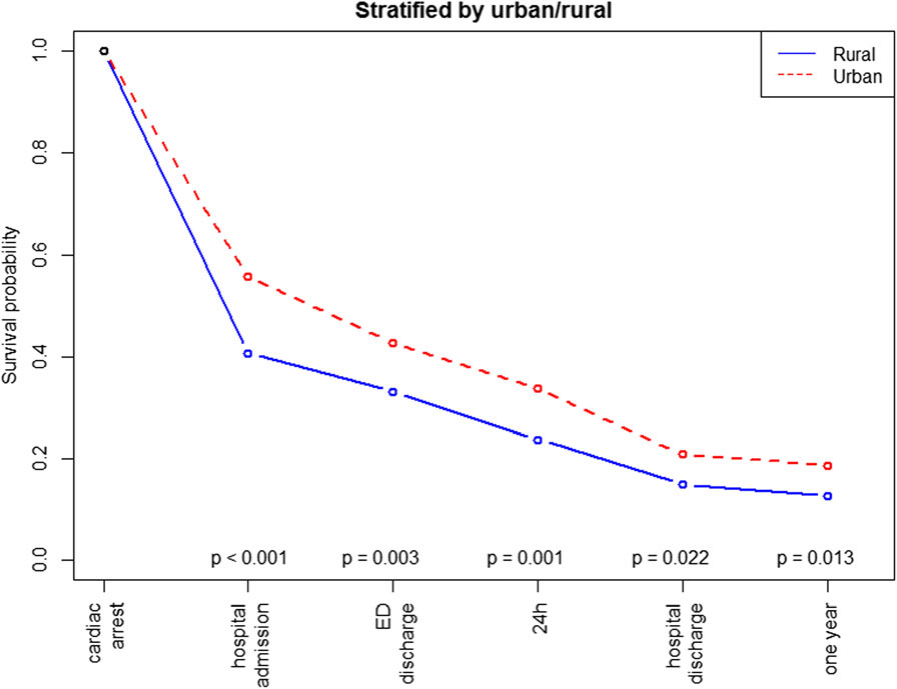
Kaplan Meier type survival curves for out-of-hospital cardiac arrest patients in rural versus urban areas. *ED* emergency department

**Fig. 3:**
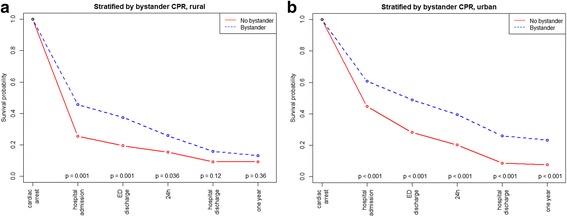
Kaplan Meier type survival curves for out-of-hospital cardiac arrest patients stratified by bystander cardiopulmonary resuscitation in rural versus urban areas. CPR cardiopulmonary resuscitation, *ED* emergency department

**Fig. 4:**
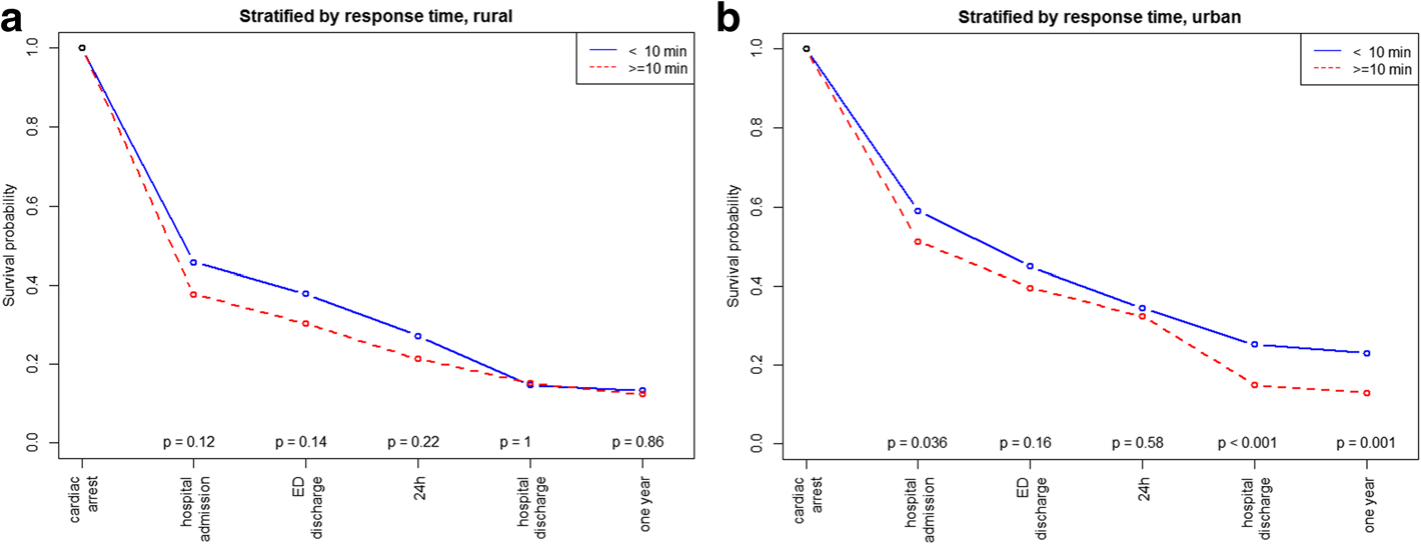
Kaplan-Meier type survival curves for out-of-hospital cardiac arrest patients stratified by emergency medical services response time in rural versus urban areas. *ED* emergency department

**Fig. 5:**
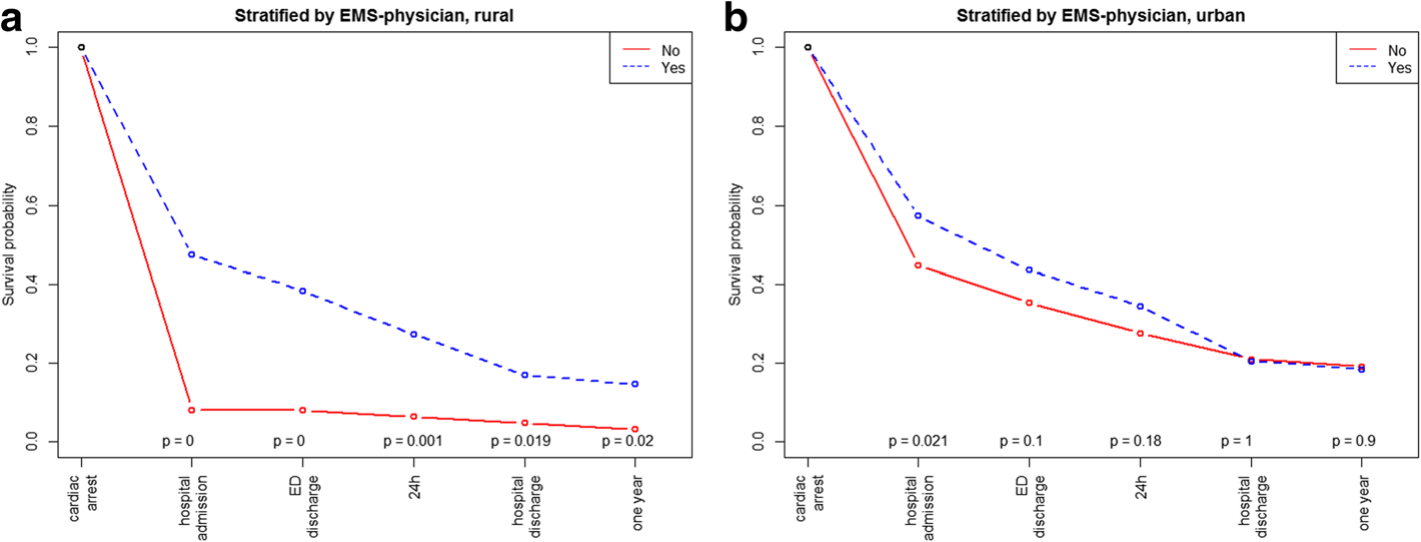
Kaplan-Meier type survival curves for out-of-hospital cardiac arrest patients, stratified by emergency medical services physician attendance in rural versus urban areas. *ED* emergency department, *EMS* emergency medical services

**Fig. 6:**
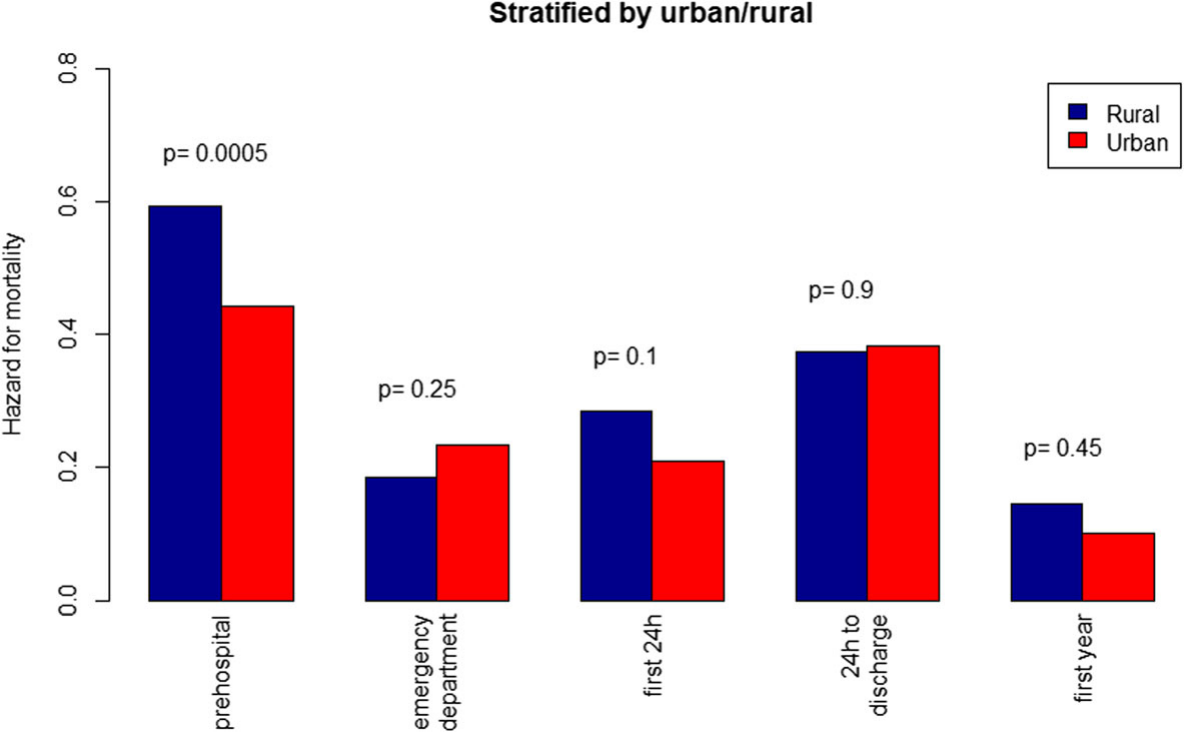
Bar graphs illustrating the hazard for mortality in out-of-hospital cardiac arrest patients in rural versus urban areas. The hazard is the probability of dying between two consecutive stages of care, given that the patient is alive at the first stage

The overall patient survival to hospital discharge was 18.8% and the unadjusted survival was higher in urban than in rural areas (Table 1). This was also reflected in the crude analysis with a significantly higher probability of survival to hospital admission (OR: 1.84, 95% CI 1.43–2.36, *p* = < 0.001), to hospital discharge (OR: 1.51, 95% CI 1.08–2.11, *p* = 0.017), and at 1 year (OR: 1.58, 95% CI 1.11–2.26, *p* = 0.012) in the urban group compared with the rural group (Table 2, Fig. 2). Bystander CPR was associated with improved survival to discharge only in urban areas (Fig. 3, *p* < 0.001). EMS response times > 10 min was associated with decreased survival to discharge only in urban areas (Fig. 4, *p* < 0.001). The attendance by an EMS physician was associated with improved survival to hospital discharge only in rural areas (Fig. 5). When adjusted for the modifiable factors listed above, the difference in survival to hospital admission, hospital discharge, and at 1 year between rural and urban areas remained (Table 3). There was a significant higher prehospital hazard of death in OHCA victims in rural areas. However, if they survived to hospital admission, this hazard difference disappeared (Fig. 6).

**Table 1:** Incidence, outcome and characteristics of medical out-of-hospital cardiac arrest patients (*n* = 1138)

	Rural area (*n* = 371)	Urban area (*n* = 767)	*p*,value	Number of missing data
OHCA incidence/100,000/ year (adjusted rate)	49 (52)	47 (56)	0.45	
Survival to hospital discharge, n (%)	55 (14.8)	159 (20.7)	0.021	0
Median patient age in years, (IQR)	69 (56–80)	70 (58–81)	0.31	1
Median EMS response time in minutes, (IQR)	11 (7–16)	9 (7–12)	< 0.001	0
Male gender, n (%)	263 (71)	522 (68)	0.37	0
Attended by EMS physician, n (%)	308 (83)	658 (86)	0.20	3
Shockable rhythm, n (%)	129 (36)	310 (41)	0.11	26
Prehospital ROSC, n (%)	111 (30)	286 (37)	0.017	0
Witnessed OHCA, n (%)	258 (70)	528 (70)	0.75	14
Bystander CPR, n (%)	267 (73)	537 (71)	0.39	11
Cardiac arrest location home, n (%)	229 (62)	508 (66)	0.17	1
Survival to hospital discharge in bystander witnessed OHCA with shockable first rhythm	43 (41)	132 (50)	0.14	0

The *p* values are calculated by Poisson regression, The Mann-Whitney test, chi-square tests as appropriate

*CPR* cardiopulmonary resuscitation, *EMS* emergency medical services, *OHCA* out-of-hospital cardiac arrest, *ROSC* return of spontaneous circulation

**Table 2:** Odds ratios of key factors associated with survival

	Survival to hospital admission			Survival to hospital discharge			1 year survival		
	OR	95% CI	*p*	OR	95% CI	*p*	OR	95% CI	*p*
Urban vs. rural	1.84	1.43– 2.36	< 0.001	1.51	1.08– 2.11	0.017	1.58	1.11– 2.26	0.012
EMS response time ≥ 10 min vs < 10 min	0.69	0.55– 0.87	0.002	0.61	0.45– 0.83	0.002	0.57	0.41– 0.79	< 0.001
Age (one additional year)	0.97	0.97 –0.98	< 0.001	0.96	0.95– 0.97	< 0.001	0.96	0.95– 0.97	< 0.001
Gender, male vs. female	1.94	1.51 –2.51	< 0.001	3.15	2.10– 4.72	< 0.001	3.02	1.98– 4.61	< 0.001
EMS physician attendance vs. no EMS-physician attendance	2.63	1.86 –3.74	< 0.001	1.37	0.87– 2.16	0.17	1.39	0.86– 2.24	0.18
Witnessed arrest vs. non-witnessed arrest	4.12	3.12– 5.44	< 0.001	7.23	4.20–12.43	< 0.001	6.63	3.78–11.61	< 0.001
OHCA location in public vs. home	1.20	1.10– 1.30	< 0.001	1.31	1.20– 1.43	< 0.001	1.27	1.16– 1.39	< 0.001
Bystander CPR vs. no bystander CPR	1.98	1.52– 2.58	< 0.001	3.05	2.00– 4.65	< 0.001	2.84	1.83– 4.39	< 0.001
Shockable vs. non-shockable rhythm	8.25	6.21– 10.95	< 0.001	25.74	15.71–42.18	< 0.001	39.52	21.14–73.87	< 0.001
Cardiac vs. medical cause for cardiac arrest	1.31	0.92– 1.88	0.14	0.34	0.18–0.63	< 0.001	0.16	0.07– 0.40	< 0.001

Odds ratios (OR) in univariable analysis of key factors associated with survival to hospital admission, survival to hospital discharge and 1 year survival in out-of-hospital cardiac arrest (OHCA) (n = 1138). *CI c*onfidence interval, *CPR* cardiopulmonary resuscitation, *EMS* emergency medical services, *OR* odds ratio

**Table 3:** Adjusted odds ratios for survival to different stages of care using geographical area and modifiable factors in the adjustments

	Survival to hospital admission			Survival to hospital discharge			1-year survival		
	OR	95% CI	*p*	OR	95% CI	*p*	OR	95% CI	*p*
OHCA in urban area vs. rural area	9.28	3.42– 25.21	< 0.001	5.11	1.45– 18.05	0.011	6.81	1.52– 30.49	0.012
Bystander CPR vs. no bystander CPR	2.02	1.54– 2.65	< 0.001	2.98	1.95– 4.56	< 0.001	2.76	1.77– 4.29	< 0.001
EMS response time ≥ 10 min vs. ≤ 10 min.	0.69	0.54– 0.88	0.003	0.61	0.44– 0.84	0.002	0.57	0.41– 0.80	< 0.001
EMS physician attendance in rural area vs. no EMS physician attendance in rural area	10.7	3.94– 26.25	< 0.001	3.58	1.07– 12.01	0.039	4.71	1.10– 20.15	0.037
EMS physician attendance in urban area vs. no EMS physician attendance in urban area	1.62	1.06– 2.48	0.025	0.91	0.54– 1.45	0.736	0.91	0.53– 1.56	0.730

Adjusted odds ratios (multivariable analysis) of modifiable factors associated with survival to different stages of care in rural and urban out-of-hospital cardiac arrest (OHCA) (n = 1138). Due to significant interaction with area EMS-physician are reported separately for urban and rural area

*CI* confidence interval, *CPR* cardiopulmonary resuscitation, *EMS* emergency medical services, *OR* odds ratio

## Discussion

We conducted this retrospective analysis of registry data including 1138 OHCA patients with attempted resuscitation in a region where a high survival rate has previously been reported [14]. We found a significantly higher probability of survival to hospital admission, to hospital discharge, and at 1 year in the urban group compared with the rural group of patients. While bystander CPR and EMS response time < 10 min were associated with a favorable outcome in urban patients at all stages of care, the attendance of an EMS physician was not. When adjusted for the modifiable factors; bystander CPR, attendance of EMS physician and EMS response time, the significant difference in survival to hospital admission, to hospital discharge, and at 1 year between rural and urban OHCA patients remained.

The present study shows a strong association between bystander CPR and survival, a finding that has been confirmed in several previous studies [5, 21]. However, the current study also indicates that bystander CPR had a significant impact on survival in urban areas only. This major finding cannot be explained by differences in initial cardiac rhythm, gender, OHCA location, witnessed cardiac arrest, or bystander CPR rate between rural and urban areas. One reason could be lack of statistical power to detect a significant effect of bystander CPR in the rural group as there are few patients alive to hospital discharge. However, when assessing this finding, context characteristics must be considered. The social structure of rural communities means that there are fewer potential CPR bystanders compared with more densely populated areas. In urban areas, a greater number of potential bystanders are present, or in proximity, to initiate efficient interventions, such as calling the EDC and providing CPR in OHCA incidents. This may affect the quality of the handling of the OHCA incident before the EMS’ arrival.

In the present study, the median EMS response time was 2 min shorter for the urban group than for the rural group. Considering the rapidly declining effect of bystander CPR with time, the lack of statistical significance between survival and EMS response time < 10 min in rural areas was surprising (Fig. 4) [6]. However, as time to intervention is crucial for survival, longer EMS response times may still explain the lower survival rate in the rural group. Optimizations in logistics can save lives, [22, 23] and EMS response time is thus one modifiable factor in improving survival.

Attendance by an EMS physician in OHCA was associated with overall improved survival at in-hospital stages of care and at 1 year in rural areas, but only with survival to hospital admission for OHCA in the urban group. Potential selection bias for EMS physician attendance at OHCA incidents may partially explain its association with short-term survival. Also, the high OR for survival to hospital discharge in rural areas may indicate a selection bias where EMS physician is called to certain types of OHCA. Some studies have been inconclusive or not shown a survival benefit of EMS physician attendance in OHCA [24, 25],. Hamilton et al. found an association between EMS physician attendance and both ROSC at the scene and 30-day survival [7]. The attendance by EMS physicians might have the greatest impact after ROSC has been achieved [26], and for the introduction of new treatment modalities, for example extracorporeal membrane oxygenation [27].

The higher OHCA survival in urban compared to rural areas found in the present study corresponds with the results of previous reports [9–11]. Still, among patients admitted alive, our results indicate no significant difference in later mortality, whether in the emergency department, 24 h after hospital admission, mortality at hospital discharge, or at 1 year. This is an important finding, and to our knowledge not reported before. It implies that further overall improvement in survival in rural areas must be based on community-based interventions [28]. Survival following rural OHCA could be improved by strengthening rural communities’ CPR training [29], increase the use of dispatcher-assisted CPR [30], implement first-responder programs [31], provision of public access defibrillators [32], and optimize the localization of EMS units [23]. With implementation of best practice, survival following OHCA could also be improved [33].

In the present study, bystander-witnessed OHCA with shockable first rhythm was 48.3% survival. Compared to a previous study conducted in the Stavanger region, in which a 52% survival to hospital discharge rate was found, the survival rate in the present study remains very good (Table 1) [14]. However, unlike the previous study, the results in the current study are based on non-EMS witnessed OHCA patients with a presumed medical cause (including a cardiac cause) for OHCA. Although the patient populations of the two studies are not entirely comparable, the results in the current study imply that the survival rate in the Stavanger region has not improved during the last decade, which contrasts with what has been found in several other regions [34, 35]. Opportunities for improvements include shortening of EMS response times, and strengthening community preparedness by e.g. additional lay person first responders via short message service alert or mobile app-based alert system. [36] To save more lives following OHCA, continuous endeavors to optimize modifiable factors are required to improve every link in the chain of survival.

### Limitations

The data in this study did not allow us to assess patient neurological status and recovery in the surviving patients. However, previous studies from the Stavanger region have shown a good neurological status for the surviving patients [14], and there have been no major changes in OHCA patient treatment to indicate that this has changed over time. We chose the population and EMS response time categories that were appropriate for our region, so this choice may not be generalizable to other regions and countries. Further, we did not record the presence or absence of dispatcher-assisted CPR, which has been shown to affect survival in OHCA [37]. The calculated incidence of 47 OHCA per 100,000 per year in our region is low compared to other reports [38], but the adjusted rate of 54 OHCA per 100,000 per year is according to OHCA in the general Norwegian population [39]. Also, for quality assurance, a designated nurse cross-checked data before entering into the OHCA database. Thus, we do not suspect selective reporting. The study region was that encompassed by the Stavanger Hospital Trust and the current findings may therefore not be generalizable to other different systems. Several hypothesis tests are conducted without any explicit adjustment for multiple testing. We acknowledge that some small *p* values might have been obtained by chance, and in particular should *p* values close to 0.05 be interpreted with care.

## Conclusions

We found that OHCA survival was higher in urban compared to rural areas, and that the impact of the prehospital modifiable factors bystander CPR, EMS response time, and EMS physician attendance differed in urban and rural areas. The main difference is due to a lower ROSC rate and hospital admission rate of OHCA patients in rural areas. Importantly, in patients admitted alive to the hospital, survival rate did not differ between rural versus urban areas. Further improvements in survival in rural areas can be built on community-based interventions such as CPR training, first-responder programs, and public access defibrillation.
